# Coronavirus disease 2019 (COVID-19) among healthcare workers: A call for a low-threshold in-hospital screening

**DOI:** 10.1017/ice.2020.324

**Published:** 2020-07-03

**Authors:** Hytham K. S. Hamid

**Affiliations:** Department of Surgery, Soba University Hospital, Khartoum, Sudan

*To the Editor—*A recent large study have shown that only 3% of healthcare workers (HCWs) infected with the novel severe acute respiratory coronavirus virus 2 (SARS-CoV-2) have been exposed to an inpatient with coronavirus disease 2019 (COVID-19) prior to the onset of symptoms, suggesting that most HCWs acquire infection in the community, perhaps due to contact with presymptomatic or asymptomatic carriers, rather than in hospital settings.^1^ The high prevalence of infection (11%–20%) among HCWs supports this assertion,^2,3^ despite the reportedly low risk of nosocomial infection associated with SARS-CoV-2.^1,4,5^

Wee et al^6^ recently reported their experience with syndromic surveillance of HCWs for COVID-19. The surveillance was based on symptoms of acute respiratory illness (ARI) and fever. Pointing to community as well as in-hospital secondary transmission, these researchers detected 14 cases of COVID-19 among HCWs with 4 clusters; most were linked via transmission outside of hospital.^6^ However, although syndromic surveillance, based on fever and ARI symptoms, was shown to be effective during the outbreak of severe acute respiratory syndrome (SARS),^7^ it may not be as effective in containment of COVID-19 infection because of different patterns of clinical presentations. Indeed, in the study by Wee et al,^6^ heat maps did not pick up the cluster with suspected intrahospital spread. Moreover, in a study by Tostmann et al^2^ employing a low-threshold screening for SARS-CoV-2, most HCWs with SARS-CoV-2 infection had mild clinical presentations, frequently not including fever or respiratory symptoms. Using the same clinical data, these researchers developed a model, excluding fever and cough, to predict COVID-19 among HCWs with a fair discriminative ability.^2^

Early identification and control of COVID-19 among HCWs is of paramount importance particularly in the postoutbreak period to prevent in-hospital secondary transmission to other HCWs and inpatients. The concern of transmitting infection into the healthcare system has been highlighted in a recent study in which HCWs were linked to transmission of COVID-19 into long-term healthcare facilities.^8^ Considering that as many as 50% of all SARS-CoV-2 infections are asymptomatic,^9^ it would seem appropriate, when resources are available, to perform routine SARS-CoV-2 nasopharyngeal screening for all HCWs. The prediction model described by Tostmann et al,^2^ rather than fever and ARI symptoms, can be used to guide a targeted screening strategy in settings with limited availability of testing materials.
